# Assessment of Population Well-Being With the Mental Health Quotient (MHQ): Development and Usability Study

**DOI:** 10.2196/17935

**Published:** 2020-07-20

**Authors:** Jennifer Jane Newson, Tara C Thiagarajan

**Affiliations:** 1 Sapien Labs Arlington, VA United States

**Keywords:** psychiatry, public health, methods, mental health, population health, social determinants of health, global health, behavioral symptoms, diagnosis, symptom assessment, psychopathology, mental disorders, mhealth, depression, anxiety, attention deficit disorder with hyperactivity, autistic disorder, internet

## Abstract

**Background:**

Existing mental health assessment tools provide an incomplete picture of symptom experience and create ambiguity, bias, and inconsistency in mental health outcomes. Furthermore, by focusing on disorders and dysfunction, they do not allow a view of mental health and well-being across a general population.

**Objective:**

This study aims to demonstrate the outcomes and validity of a new web-based assessment tool called the Mental Health Quotient (MHQ), which is designed for the general population. The MHQ covers the complete breadth of clinical mental health symptoms and also captures healthy mental functioning to provide a complete profile of an individual’s mental health from clinical to thriving.

**Methods:**

The MHQ was developed based on the coding of symptoms assessed in 126 existing Diagnostic and Statistical Manual of Mental Disorders (DSM)–based psychiatric assessment tools as well as neuroscientific criteria laid out by Research Domain Criteria to arrive at a comprehensive set of semantically distinct mental health symptoms and attributes. These were formulated into questions on a 9-point scale with both positive and negative dimensions and developed into a web-based tool that takes approximately 14 min to complete. As its output, the assessment provides overall MHQ scores as well as subscores for 6 categories of mental health that distinguish clinical and at-risk groups from healthy populations based on a nonlinear scoring algorithm. MHQ items were also mapped to the DSM fifth edition (DSM-5), and clinical diagnostic criteria for 10 disorders were applied to the MHQ outcomes to cross-validate scores labeled at-risk and clinical. Initial data were collected from 1665 adult respondents to test the tool.

**Results:**

Scores in the normal healthy range spanned from 0 to 200 for the overall MHQ, with an average score of approximately 100 (SD 45), and from 0 to 100 with average scores between 48 (SD 21) and 55 (SD 22) for subscores in each of the 6 mental health subcategories. Overall, 2.46% (41/1665) and 13.09% (218/1665) of respondents were classified as clinical and at-risk, respectively, with negative scores. Validation against DSM-5 diagnostic criteria showed that 95% (39/41) of those designated clinical were positive for at least one DSM-5–based disorder, whereas only 1.14% (16/1406) of those with a positive MHQ score met the diagnostic criteria for a mental health disorder.

**Conclusions:**

The MHQ provides a fast, easy, and comprehensive way to assess population mental health and well-being; identify at-risk individuals and subgroups; and provide diagnosis-relevant information across 10 disorders.

## Introduction

### Background

According to the World Health Organization, mental health is “a state of well-being in which the individual realizes his or her own abilities, can cope with the normal stresses of life, can work productively and fruitfully, and is able to make a contribution to his or her community” [1]. According to this definition, any framework of mental health assessment should therefore not only reflect the presence of dysfunction but also provide insight into positive aspects of mental well-being, ensuring it is applicable not only for clinical groups but also for the wider population [2]. In addition, although personalized approaches to mental health are essential in ensuring effective treatment outcomes at the individual level [3-5], population-level approaches provide an understanding of the broader geographical, cultural, and experiential factors that influence mental health on a macroscale [6,7]. This latter perspective provides an opportunity to develop interventions that induce large-scale shifts in population well-being and is becoming increasingly important for understanding how to improve mental health outcomes [8,9]. However, current approaches to mental health assessment pose considerable challenges to these goals and ideals.

### Challenges in Mental Health Assessment

One major challenge is that the clinical heritage of mental health assessment means that most tools are not designed for the general population but are instead built around specific psychiatric disorder categories based on the clinical classification systems of the Diagnostic and Statistical Manual of Mental Disorders (DSM) [10] or the International Classification of Diseases (ICD) [11]. In this way, an assessment can identify whether an individual exhibits symptoms pertaining to a specific mental health disorder such as depression, attention-deficit/hyperactivity disorder (ADHD), or alcohol addiction but does not readily provide a perspective of their overall mental health. In contrast, the general population falls along a continuum ranging from disordered to thriving and therefore having a system that is predominantly focused on disorders and dysfunction, without an equivalent understanding of well-being, presents a challenge to advancing the understanding of the borders between normal mental health and clinical disorder [12-15], especially because many mental health symptoms such as sadness, anxiety, and risk-taking also fall within the spectrum of normal mental functioning in the general population. Understanding when such normal mental functions cross the boundary to become symptoms requires an assessment approach that is designed for the general population and that encompasses the range from clinical dysfunction to positive mental assets.

A second challenge is that existing mental health assessment tools, despite being broadly based on symptom criteria defined by DSM or ICD classification systems, are highly heterogeneous. Our recent analysis of 126 commonly used mental health screening assessments revealed considerable inconsistency in symptom assessment across different tools focusing on the same disorder and substantial overlap between disorders [16]. Consequently, two assessments that target the same population group, but which used different tools to assess their experience of mental health problems, may deliver different results because they are assessing a different set of symptoms (see also the study by Fried [17]). This creates ambiguity, bias, and inconsistency in mental health determination and confuses the development of effective treatments and interventions to promote well-being within the general population. Moreover, when examining assessment tools that span multiple disorders and therefore aim to provide a broader perspective on mental health, Newson et al [16] found that none of the 16 cross-disorder assessment tools that were analyzed covered the complete breadth of mental health symptoms and few considered positive mental assets (see also the study by Allsopp et al [18]). This suggests that existing cross-disorder tools fail to provide a complete picture of mental health symptoms and positive assets that would apply to both clinical and normal healthy populations.

### The Mental Health Quotient

To address these challenges, we have developed a new web-based assessment tool called the Mental Health Quotient (MHQ) [19], which is designed for the general population and covers the complete breadth of clinical mental health symptoms as well as positive mental assets. It has been developed based on an extensive review of the way mental health is assessed in clinical and research fields [16], and its purpose is to provide a comprehensive assessment of an individual’s mental health profile ranging from clinical to thriving, which is suitable for both clinical and population-based assessments. Here, we describe the development of the MHQ and provide preliminary data from a cross-section of the population to illustrate its output.

## Methods

### Design and Development of the MHQ

#### Key Design Criteria

The key design criteria of the MHQ were that it had to be fast and easy to complete by the general population (take ≤15 min) and administered such that respondents felt confident in providing honest responses that were reflective of the current perception of the respondent’s mental health. The MHQ was therefore designed to provide a view of respondent perception within their individual life context, which is not absolute, that is, what one person means by a severity rating of 8 could be different from what someone else means in actual life outcomes and can change over time. This is in line with how the majority of mental health symptoms are typically assessed. In addition, as an output, it would have to provide an overall score of mental health as well as scores along key macro dimensions. Taking these requirements into consideration, the standard version of the MHQ was developed to be taken on the web anonymously and provide a score and full individual report that encourages honest self-report.

#### Developing a Complete Inventory of Mental Health and Well-Being Elements

**Figure 1:**
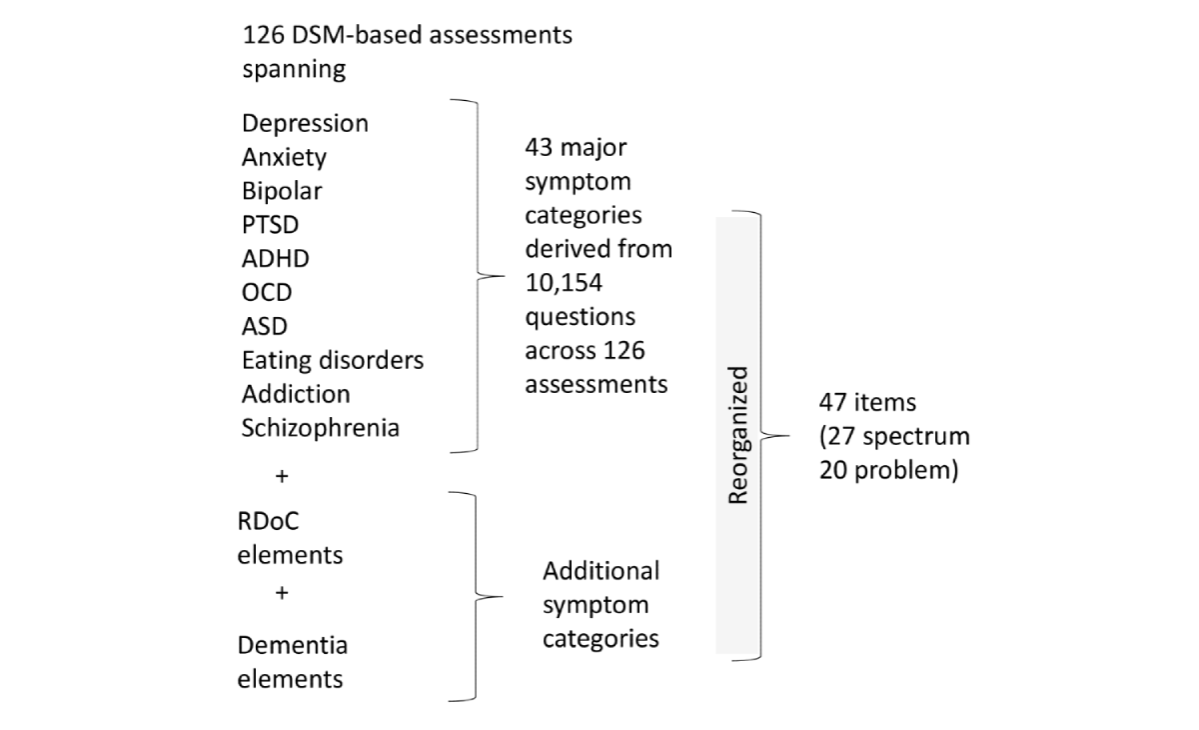
Diagram illustrating the method of development of the Mental Health Quotient. A total of 126 commonly used psychiatric assessment tools covering 10 disorders (as well as those taking a cross-disorder approach) were reviewed and consolidated into 43 symptom categories. These categories, together with additional symptom categories taken from a review of Research Domain Criteria constructs as well as dementia elements, were reorganized into a final set of 47 items that were divided into spectrum and problem items for inclusion in the Mental Health Quotient. ADHD: attention-deficit/hyperactivity disorder; ASD: autism spectrum disorder; DSM: Diagnostic and Statistical Manual of Mental Disorders; OCD: obsessive-compulsive disorder; PTSD: post-traumatic stress disorder; RDoC: Research Domain Criteria.

The MHQ was developed based on a comprehensive review of symptoms assessed across 126 commonly used psychiatric assessment tools (Figure 1), spanning disorders of depression, anxiety, bipolar disorder, ADHD, post-traumatic stress disorder (PTSD), obsessive-compulsive disorder (OCD), addiction, schizophrenia, eating disorder, and autism spectrum disorder (ASD), and cross-disorder tools (see the study by Newson et al [16] for a complete list of assessment tools).

A total of 10,154 questions, taken from these 126 assessment tools, were identified and coded based on a judgment of their semantic content and consolidated into a set of 43 symptom categories by grouping similar preliminary symptom codings (see the study by Newson et al [16] for a more detailed description). This approach was selected because diagnoses are determined from self-reported symptoms that are based on a semantic description, rather than underlying biological factors. Therefore, the objective was not to reduce the scale down into independent items in terms of occurrence but to cover the breadth of symptoms of mental health assessment based on their semantic description (as an example of this approach, fever and fatigue are semantically distinct but often co-occur). This set of symptom categories was then reviewed in the context of the Research Domain Criteria (RDoC) constructs and subconstructs put forward by the National Institute of Mental Health [20-22], and a few additions were made to ensure that the list of items reflected the components within this non-DSM framework. Next, we ensured that there were items within the MHQ that reflected symptoms of neurological disorders (eg, dementia) that were not covered in the original review [16]. The resulting categories were then restructured as follows. First, categories that reflected purely physical symptoms (eg, urination problems) were consolidated under the generalized item of Physical health issues. Second, categories that reflected items that a naive respondent might find difficult to differentiate (eg, delusions and unwanted thoughts) were also consolidated. Third, where a category reflected multiple symptoms or functions, it was split into 2 (or 3) independent items to make it clear to the respondent which function or symptom was being assessed (eg, sleep quality vs nightmares). This resulted in 47 semantically distinct items (Textbox 1).

The resultant items from this review and reorganization were then split into 2 formats: those mental functions that could manifest as a spectrum from positive to negative, which we called spectrum items (27 questions in all), and those symptoms that purely represented detractions from overall mental health, which we called problem items (20 questions in all).

**List of spectrum and problem items.**

Spectrum questions

- Adaptability to change
- Self-worth and confidence
- Creativity and problem solving
- Drive and motivation
- Stability and calmness
- Sleep quality
- Self-control and impulsivity
- Ability to learn
- Coordination
- Relationships with others
- Emotional resilience
- Planning and organization
- Physical intimacy
- Speech and language
- Memory
- Social interactions and co-operation
- Decision making and risk-taking
- Curiosity, interest, and enthusiasm
- Energy level
- Emotional control
- Focus and concentration
- Appetite regulation
- Empathy
- Sensory sensitivity
- Self-image
- Outlook and optimism
- Selective attention

Problem questions

- Restlessness and hyperactivity
- Fear and anxiety
- Susceptibility to infection
- Aggression toward others
- Avoidance and withdrawal
- Unwanted, strange, or obsessive thoughts
- Mood swings
- Sense of being detached from reality
- Nightmares
- Addictions
- Anger and irritability
- Suicidal thoughts or intentions
- Experience of pain
- Guilt and blame
- Hallucinations
- Traumatic flashbacks
- Repetitive or compulsive actions
- Feelings of sadness, distress, and hopelessness
- Physical health issues
- Confusion or slowed thinking

#### Question Format

**Figure 2:**
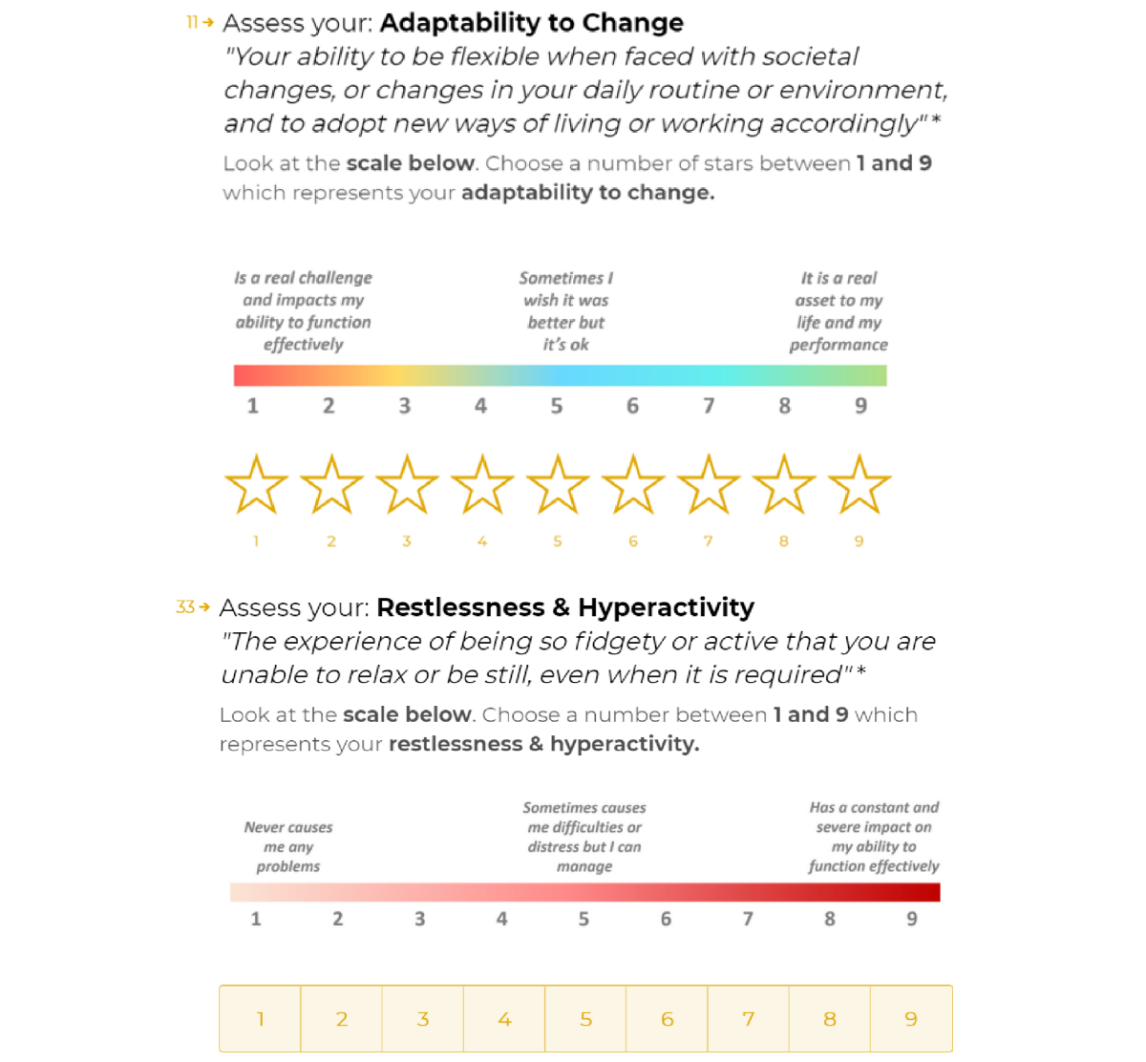
Example of a spectrum question and a problem question. Each question was composed of an item category and a 1-sentence description of that item as well as a 1 to 9 rating scale with reference labels.

Questions were answered based on the current perception of the respondent (“Please choose your answers based on your current perception of yourself”) and were formulated on a 9-point scale reflecting the consequence on one’s life functioning and performance. Figure 2 shows an example of a spectrum question from the MHQ (on adaptability to change) and an example of a problem question. Each question included a broad category label as well as a one-sentence description of the item for clarity.

The scale of spectrum questions was designed to reflect functions that could be an asset for some individuals but a problem for others. In this way, spectrum questions were developed such that they did not relate to the presence or absence of a function or symptom but instead focused on the impact that the item had on the individual across a range of positive or negative possibilities. In the 9-point scale for spectrum items, 1 referred to “Is a real challenge and impacts my ability to function effectively,” 9 referred to “It is a real asset to my life and my performance,” and 5 referred to “Sometimes I wish it was better, but it’s ok.”

Problem questions were designed to reflect functions or dysfunctions that typically had a negative impact on someone’s life and could rarely be seen as a positive asset. Here, 1 on the 9-point scale referred to “Never causes me any problems,” 9 referred to “Has a constant and severe impact on my ability to function effectively,” and 5 referred to “Sometimes causes me difficulties or distress but I can manage.”

Within the spectrum and problems sections of the assessment tool, questions were presented in a random order so as not to be leading or priming for the subsequent question.

#### Demographic, Experiential, and Momentary Questions

Questions designed to collect demographic, experiential, and momentary information were also included in the MHQ assessment. These questions aimed to provide insight into the life context and situation of the individual at the time of taking the assessment to understand how they influence mental health. Demographic questions were included to ask about the nature of a person’s daily occupation, geography, age, and gender. Momentary assessments were designed to determine certain aspects of the individual’s situation, as well as their physical and mental state at the time of taking the assessment, including alertness; mood; hours slept the previous night; time since last meal; and any current physical symptoms such as headache, nausea, or pain. Experiential questions were included to ask about life satisfaction, life trauma, whether they had a diagnosed medical disorder, or whether they were currently seeking mental health treatment. These questions were answered using multiple-choice answer options, using 9-point rating scales, or using a text box, depending on the specific question type, and were included to identify how these factors influence mental health and well-being.

### Scoring of the MHQ

#### Computing the MHQ

The MHQ was not computed as a simple average of raw scores, given (1) there are both negative and positive aspects, (2) there are differences in the seriousness of consequences of different symptom types, and (3) consequences do not necessarily increase linearly at higher values on the scale. Therefore, the raw scores were transformed in 2 steps, which included a threshold-based rescaling of the 9-point scale to a positive-negative scale, followed by the application of a differential nonlinear weighting of the negative scores to better distinguish at-risk populations.

For all questions, a value *N* was determined as the rescaling threshold to separate the scale into a positive side depicting a normal range and a negative side indicating clinical risk. For problem questions, responses on the rating scale were transformed to *N – (rating response),* where *N* was a threshold number between 2 and 6 that was selected depending on the seriousness of the particular symptom and determined where the scale split between positive and negative. Thus, if *N* was 2, a rating response of 1 (representing the absence of the problem) would be rescaled to a 1, and a rating response of 9 (representing a constant and severe impact on the ability to function effectively) would be rescaled to −7. If *N* was 4, a rating response of 1 would be rescaled to 3, and a rating response of 9 would be rescaled to −5. For spectrum questions, the scores were rescaled as *(rating response) – N,* where *N* was a number between 2 and 6. Thus, if *N* was 3, a rating response of 1 (representing a constant and severe impact on the ability to function effectively) would be rescaled to a −2, and a rating response of 9 (representing an asset to life and performance) would be rescaled to 6. The specific values of *N* form part of a proprietary MHQ algorithm, where lower numbers depict items that have a greater negative consequence either to the individual or those around them when experienced at severe levels (eg, suicidal thoughts or intentions and aggression toward others). In contrast, higher *N* values depict items that were evaluated as having a less negative consequence to the individual or which are often found within a healthy population (eg, guilt and blame and adaptability to change).

After this positive-negative rescaling, a differential nonlinear weighting was applied to negative scores of different symptoms to create greater distinction in the at-risk group. This weighting value also forms part of the proprietary MHQ algorithm and, similar to *N,* was determined based on an evaluation of the negative consequence of each symptom. For example, a rescaled negative score of −7 for suicidal thoughts or intentions would be weighted more negatively than a −7 for restlessness and hyperactivity and therefore result in a greater negative amplification of the MHQ score. Similarly, a rescaled negative score of −2 for energy levels would be weighted more negatively than a −2 for creativity and problem solving and result in a greater negative amplification of the MHQ score.

The resulting rescaled and nonlinearly weighted scores across all problem and spectrum items were then summed to provide an aggregate intermediate score. This intermediate score could be either a negative or positive score, where negative scores identified those respondents who had or were at risk for a clinical mental health issue and positive scores represented a normal or healthy range of mental health. To compute the MHQ, positive scores were then normalized to a scale between 0 and 200, whereas negative scores were normalized across a smaller window of −1 to −100. The negative scale was chosen to be smaller to provide a mitigated number to minimize any psychological distress that could be induced by receiving a highly negative score. Thus, the overall MHQ score spans a possible range from −100 to +200, where negative scores reflect clinical or clinically at-risk populations and positive scores reflect the distribution of the normal healthy population. This score range was also chosen to be similar to the way that IQ scores are computed, where scores are centralized around 100.

#### MHQ Subscores

Subscores were also computed for 6 broad subcategories of mental health: core cognition, complex cognition, mood and outlook, drive and motivation, social self, and mind-body (Textbox 2).

To compute the subcategory scores, a weighted average of items for each subcategory was calculated by weighting spectrum or problem items core to the subcategory as *1* and spectrum or problem items secondary to the subcategory as *0.5*. This weighting algorithm was developed based on a review of cognitive and neuroscience models of brain functioning and forms a part of the proprietary MHQ algorithm. For example, the item *stability and calmness* was coded with a primary *1* weighting in the mood and outlook subcategory and a secondary *0*.5 weighting in the mind and body subcategory to reflect its dual components of emotion and physiological response, whereas the item *unwanted, strange, or obsessive thoughts* was dual coded with a primary weighting in the core cognition subcategory and a secondary weighting in the mood and outlook subcategory to reflect both the cognitive and emotional elements of this item. In this regard, an item could be assigned to 2 different subcategories and occasionally to 3 different subcategories. Overall, each subcategory comprised 10 to 24 items. The subcategory scores were then normalized to constrain them to a smaller scale than the overall MHQ to distinguish them from the overall score. Positive scores were normalized to the range of 0 to 100, whereas negative scores were normalized to the range of −1 to −50.

**Descriptions of the 6 subcategories of mental health.**

**Core cognition**

- The ability to function effectively and independently on a moment-to-moment basis. It includes brain functions such as attention, memory, learning, and self-control. Abnormal aspects of core cognition include severe or extreme forms of mental confusion, obsessive thoughts, sensory sensitivity, compulsive behaviors, psychosis, and hallucinations

**Complex cognition**

- The ability to synthesize and make sense of complex sets of events and situations and display a longer-term perspective in thoughts and behavior. It includes brain functions such as decision making, creativity, problem solving, planning, and adaptability to change. Abnormal forms of complex cognition are associated with extreme risk-taking and severe intolerance to change

**Mood and outlook**

- The ability to manage and regulate emotions effectively and encompasses feelings of distress like fear, anxiety, anger, irritability, guilt, and sadness. It also includes the ability to have a constructive or optimistic outlook for the future. Abnormal forms of emotional functioning include uncontrollable crying, night terrors, severe temper outbursts, extreme phobias, uncontrollable panic attacks, highly traumatic flashbacks, intense mania, or suicidal intentions

**Drive and motivation**

- The ability to work toward desired goals and to initiate, persevere, and complete activities in daily life. It is associated with interest, curiosity, and motivation and is also related to overall energy levels. Abnormal forms of drive and motivation include severe addictions that cause harm or extreme withdrawal from activities or social interaction

**Social self**

- The ability to interact with, relate to, and see oneself with respect to others. It includes factors like confidence, communication skills, self-worth, body image, empathy, and relationship building. Abnormal forms of social functioning include excessive unprovoked aggression, a strong sense of being detached from reality, or suicidal intentions

**Mind-body**

- The regulation of the balance between mind and body to ensure that any mental concerns do not manifest themselves as physical symptoms in the body in a chronic or severe way. It includes functions like sleep, appetite, coordination, physical intimacy, and fatigue. Abnormal forms of mind-body balance can include insomnia or chronic and severe pain, as well as a propensity for infection or frequent physical symptoms (eg, digestive issues) with no obvious physical cause

### Mapping of the MHQ Against DSM-5 Criteria

Given that the MHQ items were derived from validated DSM-based assessments and span the breadth of symptoms assessed across 10 DSM-derived disorders, they can be readily mapped to DSM criteria. Thus, to determine the diagnostic status in relation to the MHQ score ranges, each of the 47 MHQ question items was first mapped to the diagnostic criteria of 10 mental health disorders (depression, bipolar disorder, anxiety, OCD, PTSD, schizophrenia, eating disorder, addiction, ADHD, and ASD), as defined by the DSM fifth edition (DSM-5). For example, the MHQ items of *feelings of sadness, distress, and hopelessness* and *outlook and optimism* were mapped onto the *depressed mood* criteria for depression, whereas the MHQ items of *unwanted, strange, or obsessive thoughts*, *self-control and impulsivity*, and *emotional control* were mapped onto the *obsession* criteria for OCD. Those below the negative threshold *N* on the spectrum rating scale and above the negative threshold *N* on the problem rating scale were considered to meet the severity criteria of the DSM-5.

To arrive at the diagnostic indication, we then applied the diagnostic criteria of the DSM-5 for these 10 disorders to the MHQ responses. These criteria stated the type of symptom (eg, interest, fear), the number of symptoms required (eg, must have at least three), and whether any specific symptoms must be present (eg, depression must have either a depressed mood or markedly diminished interest) for a diagnosis of a clinical disorder. Together, this provided a view of (1) the percentage of symptoms for a particular disorder that the individual exhibits (ie, the number of severe symptoms associated with that disorder they report divided by the total number of symptoms associated with that disorder), (2) the percentage of an individual’s symptoms associated with each of the 10 DSM-5–based disorder classifications (ie, the number of severe symptoms they exhibit associated with that disorder divided by the total number of severe symptoms they report), and (3) a diagnostic indication for each disorder based on criteria-derived algorithms. An example of the MHQ output for the DSM-5 mapping of symptoms for one individual is shown in Table 1.

We note that the diagnosis is based on criteria of symptom severity but excludes specifics of frequency and duration of symptoms not captured in the MHQ, which are sometimes part of the DSM-5 diagnostic criteria.

**Table 1:** Example Mental Health Quotient output for Diagnostic and Statistical Manual of Mental Disorders, Fifth Edition mapping across 10 different mental health disorders for one individual.

Disorder	Disorder symptoms, n/N (%)	Individual’s symptoms (n=20), n (%)	Diagnostic indication
Depression	10/14 (71)	10 (50)	Positive
Anxiety	6/11 (55)	6 (30)	Negative
Bipolar	11/15 (73)	11 (55)	Negative
PTSD^a^	9/20 (45)	9 (45)	Negative
OCD^b^	4/6 (67)	4 (20)	Negative
Schizophrenia	4/7 (57)	4 (20)	Negative
Eating disorder	3/3 (100)	3 (15)	Positive
Addiction	1/4 (25)	1 (5)	Negative
ADHD^c^	4/8 (50)	4 (20)	Negative
ASD^d^	2/9 (22)	2 (10)	Negative

^a^PTSD: post-traumatic stress disorder.

^b^OCD: obsessive-compulsive disorder.

^c^ADHD: attention-deficit/hyperactivity disorder.

^d^ASD: autism spectrum disorder.

### Reporting of the MHQ

**Figure 3:**
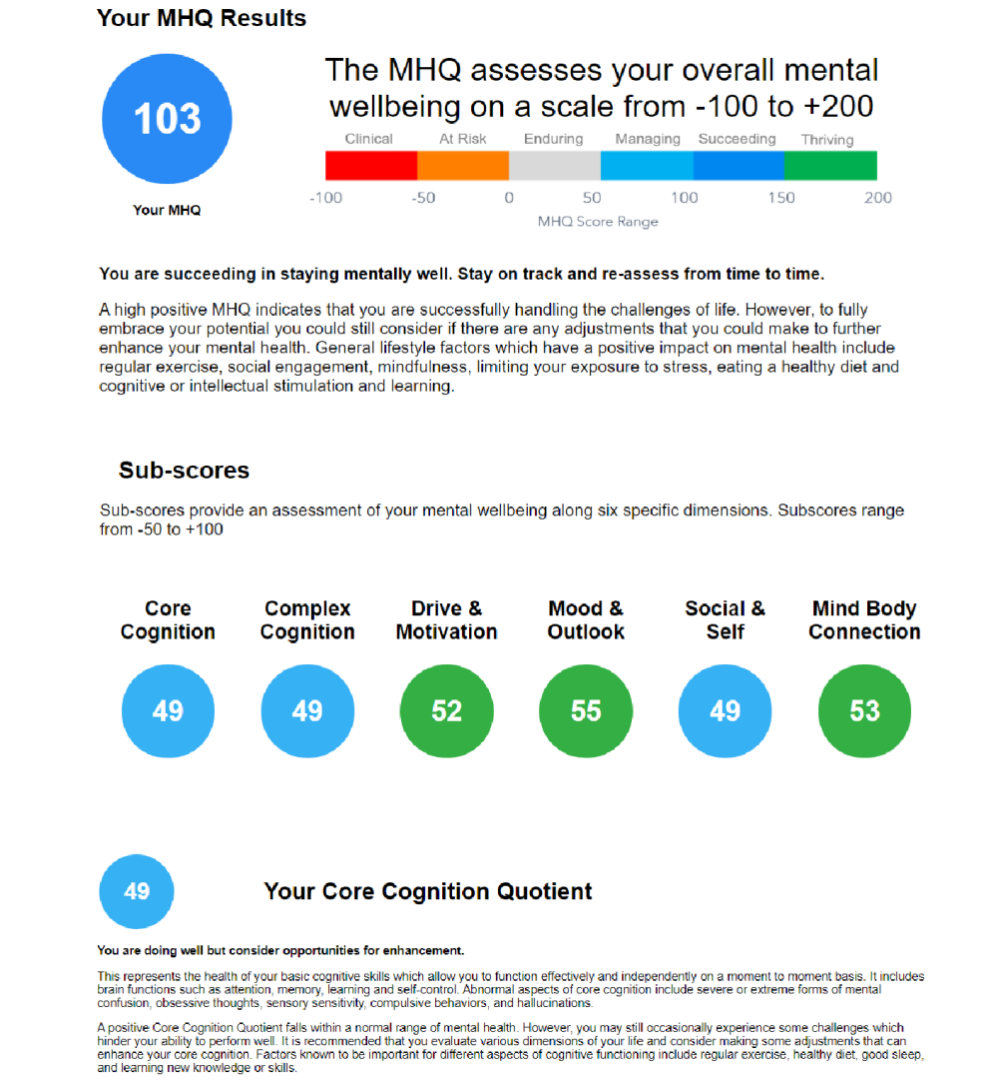
Extract of an example Mental Health Quotient results report. The report details the overall Mental Health Quotient score and recommendations based on that score. It also details each of the 6 subcategory scores as well as descriptions and recommendations based on each of those subcategory scores. MHQ: Mental Health Quotient.

The output of the MHQ was summarized both as scores as well as into an optional detailed report with recommendations for action that could be obtained by the respondent. Providing a detailed report ensured greater interest of the respondent to answer questions thoughtfully and accurately. Figure 3 shows an extract of an example MHQ results report detailing the MHQ score and subscores. The first section offers an overall MHQ score and a recommendation based on that score. The following sections offer scores for each of the 6 subcategories (Textbox 2) and recommendations based on each of those scores.

DSM-5-based mapping (eg, as shown in Table 1) is not included in the current iteration of the individual output report, although it may be included in the future. When the MHQ is used in a clinical setting, for instance, the DSM-5 mapping can be provided to an individual’s physician to provide transdiagnostic insight.

## Results

### Testing of the MHQ in the General Population

#### Participant and Protocol for Data Collection

A total of 1961 responses were collected in the study. Respondents were recruited from the websites of Psychology Today and Sapien Labs using a series of blog articles targeted at adults from July 2019 to February 2020 by providing links to the study. The study received ethics approval from the Health Media Lab Institutional Review Board. Respondents took part by accessing the MHQ on the web [19] and completing the assessment. Those aged younger than 18 years were not eligible to participate. On average, the assessment took 14 min to complete, with the typical time taken for completion being between 8 min and 20 min (1315/1961, 67.06% of respondents). In addition, 97.96% (1921/1961) of those taking part said that the assessment was easy to understand.

Data Cleaning and Exclusion Criteria

The following exclusion criteria were applied to the responses for data cleaning purposes. First, the exclusion of all but the first of multiple assessments from the same internet protocol address. Second, those respondents who took under 7 min (an indication that the questions were not actually read) or over 1 hour to complete the assessment (suggesting that the individual was not focused on the response) were excluded. Third, exclusion of individuals who found the assessment hard to understand (ie, responded *no* to the question, “Did you find this assessment easy to understand?”). Fourth, respondents who made unusual or unrealistic responses (eg, those who stated they had not eaten for 16+ hours or who stated that they had slept for >16 hours) were excluded. We reasoned that while one might sleep longer than 16 hours or fast for a day or more under unique circumstances, these responses might be considered to be entered under distressed circumstances where thinking is physiologically impaired and therefore invalid. This resulted in the exclusion of 15.09% (296/1961) of responses (Table 2), and 1665 responses were available for the final analysis.

**Table 2:** A breakdown of the percentage of responses excluded for each exclusion criterion (N=1961).

Exclusion criteria	Responses excluded, n (%)
Repeat responses from the same respondent	56 (2.85)
Time to complete <7 min (range 2-7 min)	123 (6.27)
Time to complete >1 hour (range 1-23 hours)	47 (2.39)
Poor understanding of assessment	40 (2.04)
Over 16 hours since their last meal (range 17-52)	39 (1.98)
Over 16 hours sleep the previous night (range 31-85)	10 (0.51)

#### Respondent Profile

Overall, 61.14% (1018/1665) of the respondents were female, 36.58% (609/1665) were male, and 1.08% (18/1665) responded as a nonbinary or third gender. In addition, 1.20% (20/1665) of the respondents preferred not to reveal their gender. The age distribution of respondents ranged from 18 years to 65 years and above, with the highest number in the 25 to 34 years age bracket (444/1665, 26.66%). Only 7.02% (117/1665) of the respondents were aged 65 years and above. These specific age ranges were selected to reflect major life periods above the age of 18 years. For example, 18 to 24 years reflects early adulthood and a period when many people are students, single, and are unlikely to have children, whereas 65 years and above reflects the age at which many people retire from work.

Respondents from 90 different countries completed the survey. The majority of the respondents were from the United States (797/1665, 47.87%), whereas a notable proportion of respondents were from the United Kingdom (149/1665, 8.94%), Canada (103/1665, 6.18%), and India (86/1665, 5.16%).

#### Overall MHQ Scores

**Figure 4:**
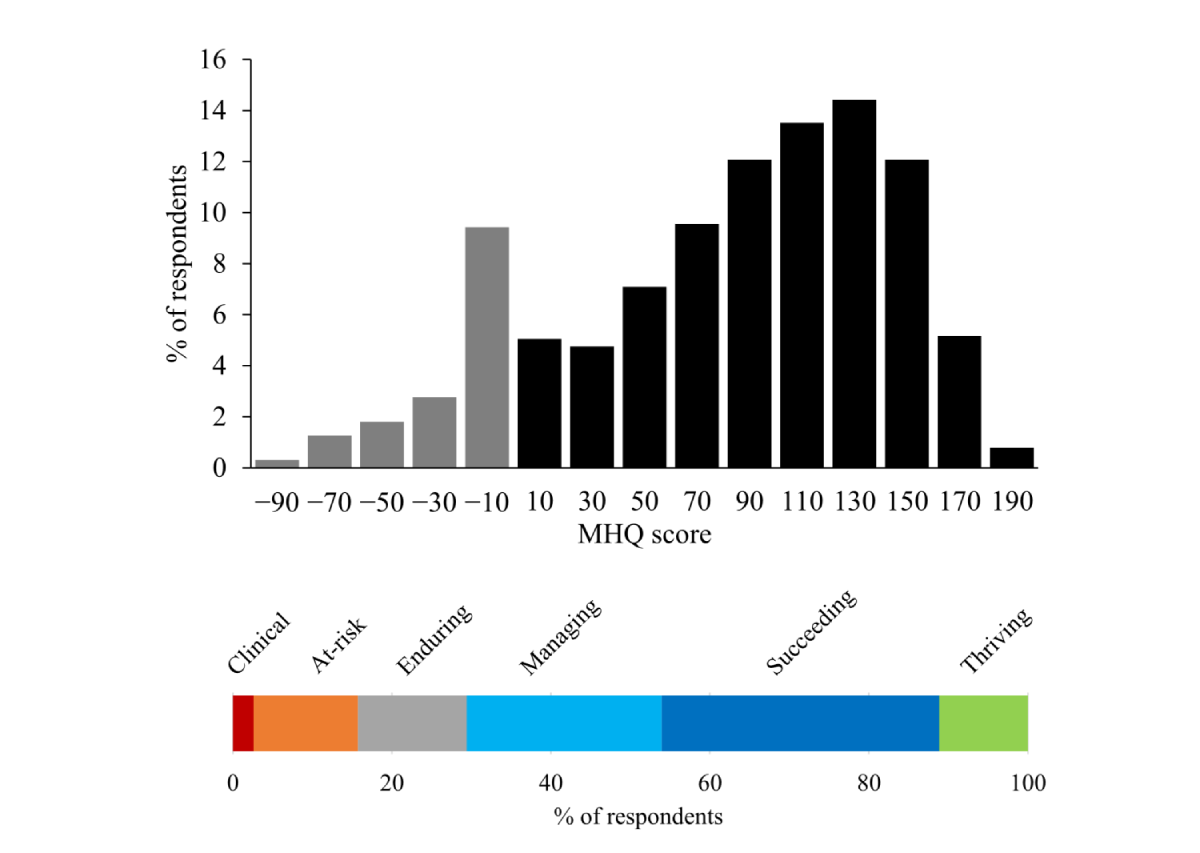
Distribution of Mental Health Quotient scores across 1665 respondents. Shows the percentage of respondents falling into Mental Health Quotient score windows ranging from −100 to +200 and across each of the 6 MHQ score levels. Gray bars denote negative scores and black bars denote positive scores. MHQ score levels are (from left to right) clinical (score range: −100 to −51), at-risk (−50 to −1), enduring (0-50), managing (51-100), succeeding (101-150), and thriving (151-200). MHQ: Mental Health Quotient.

First, we examined the overall MHQ scores across 1665 respondents. These scores ranged from −99 to +191 (on a scale of −100 to +200), where 84.44% (1406/1665) of scores fell within the positive or normal healthy range and 15.55% (259/1665) fell within the negative range indicating clinical risk. The distribution is shown in Figure 4. The overall MHQ scores had an average of 81 (median 94 and mode 139), while the positive MHQ scores had an average of 101 (median 105 and mode 139) and the negative MHQ scores had an average of −24 (median −15 and mode −4). To obtain an interpretative picture of these scores, we further grouped MHQ scores into 6 levels according to their score window (Figure 4). In the positive score range, +151 to +200 was labeled as thriving (184/1665, 11.05% of the respondents), +101 to +150 was labeled as succeeding (581/1665, 34.89% of the respondents), +51 to +100 was labeled managing (417/1665, 25.04% of the respondents), and 0 to +50 was labeled enduring (224/1665, 13.45% of the respondents). In the negative range, 13.09% (218/1665) of the respondents fell in the −˗1 to −50 score range labeled at-risk for a mental health disorder, whereas 2.46% (41/1665) of respondents fell in the −51 to −100 range, representing those who would likely require immediate clinical intervention (labeled clinical). The proportion of respondents reporting negative scores is therefore in line with the annual prevalence rates of mental health disorders reported from other sources [23-25].

**Figure 5:**
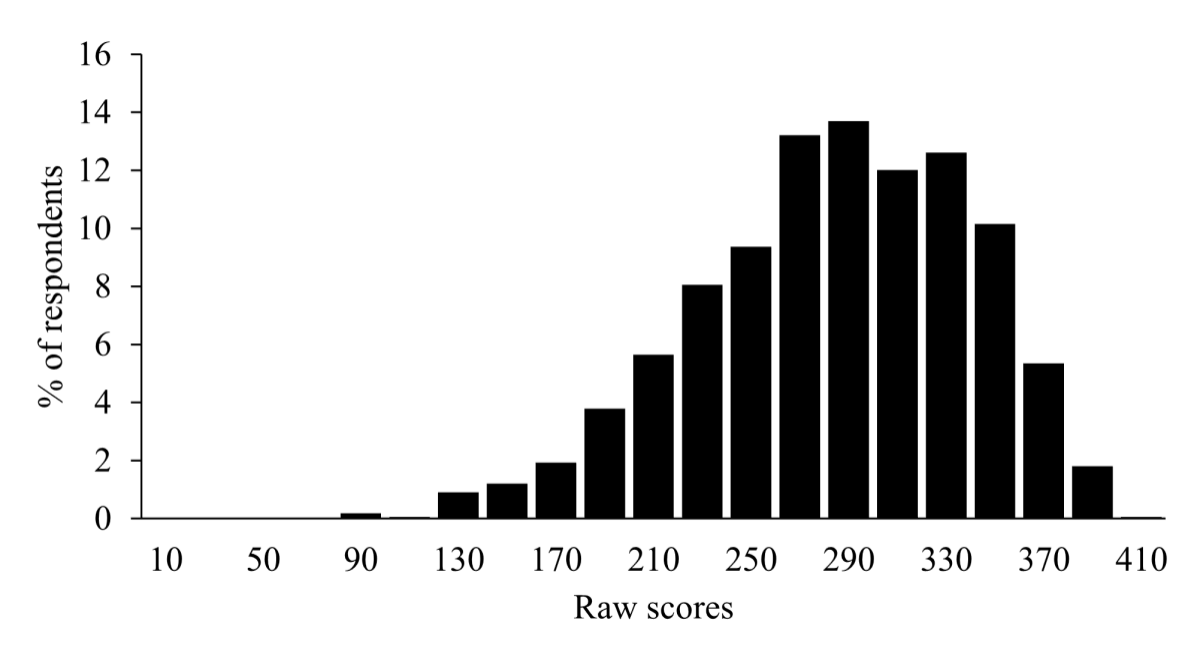
Distribution of raw scores depicting the percentage of respondents falling into different raw score brackets. Raw scores were calculated as the sum of spectrum question rating responses and reverse-scored problem question rating responses (ie, where 1 is converted to a 9 and vice versa to maintain a consistent positive-negative direction).

There were certain important characteristics of the MHQ score distribution. First, the scale spanned both positive and negative numbers, and the distribution was more heavily skewed to the left compared with a simple average of the raw scores (Figure 4 in comparison with Figure 5). This reflects the characteristics of the algorithm (negative thresholding and nonlinear weighting, see section Scoring of the MHQ), which creates a greater distinction between people who have negative symptoms of different levels of seriousness and life consequence. Second, there was a peak in the negative range in the bin immediately to the left of 0. This arises because of the compression of the negative scores to a smaller scale of 50% of the positive scale, such that each bin would be double what it would otherwise be. The rationale for this differential was to mitigate stress to the respondent.

#### Validation of MHQ Score Labels Against DSM-5 Diagnostic Criteria

To determine the validity of the MHQ scoring approach, we applied DSM-5–mapped diagnostic criteria from 10 different mental health disorders to the MHQ responses (see the Methods section). This rule-based algorithm identified respondents who met the criteria for a diagnosis of at least one mental health disorder, out of a possible 10 mental health disorders. We then examined the pattern of diagnoses across the different MHQ levels from clinical to thriving. We found that 95% (39/41) of individuals with an MHQ score in the clinical range met the diagnostic criteria for at least one mental health disorder, and 30.7% (67/218) of those in the at-risk range met the diagnostic criteria for at least one mental health disorder. Those in the clinical and at-risk categories who did not meet the DSM-5 criteria for a disorder diagnosis nonetheless had a large number of severe symptoms that spanned multiple disorders (an average of 6 severe symptoms compared with an average of 1 in the positive MHQ score group).

Within the positive score range (from 0 to 200), only 1.14% (16/1406) of the respondents met the DSM-5 criteria equivalent to a disorder diagnosis, with 88% (14/16) of these being in the enduring category. Thus, MHQ scores exhibit both a low false-positive rate within the clinical score range and a low false-negative rate within the positive score range.

#### MHQ by Age and Gender

**Figure 6:**
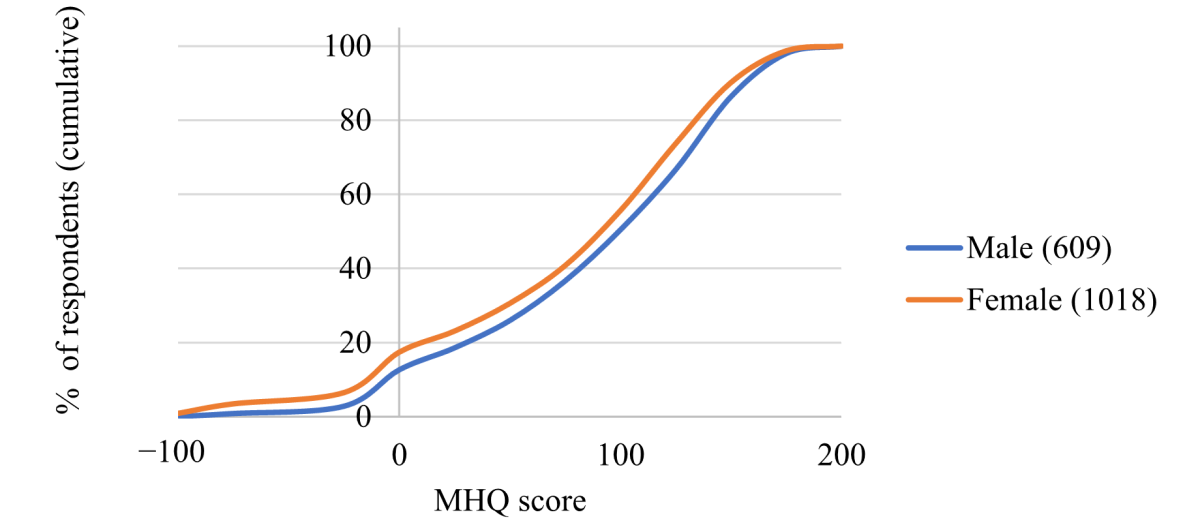
Cumulative percentage of respondents across the Mental Health Quotient score range for male and female groups (N values for male and female groups shown in the legend). MHQ: Mental Health Quotient.

**Figure 7:**
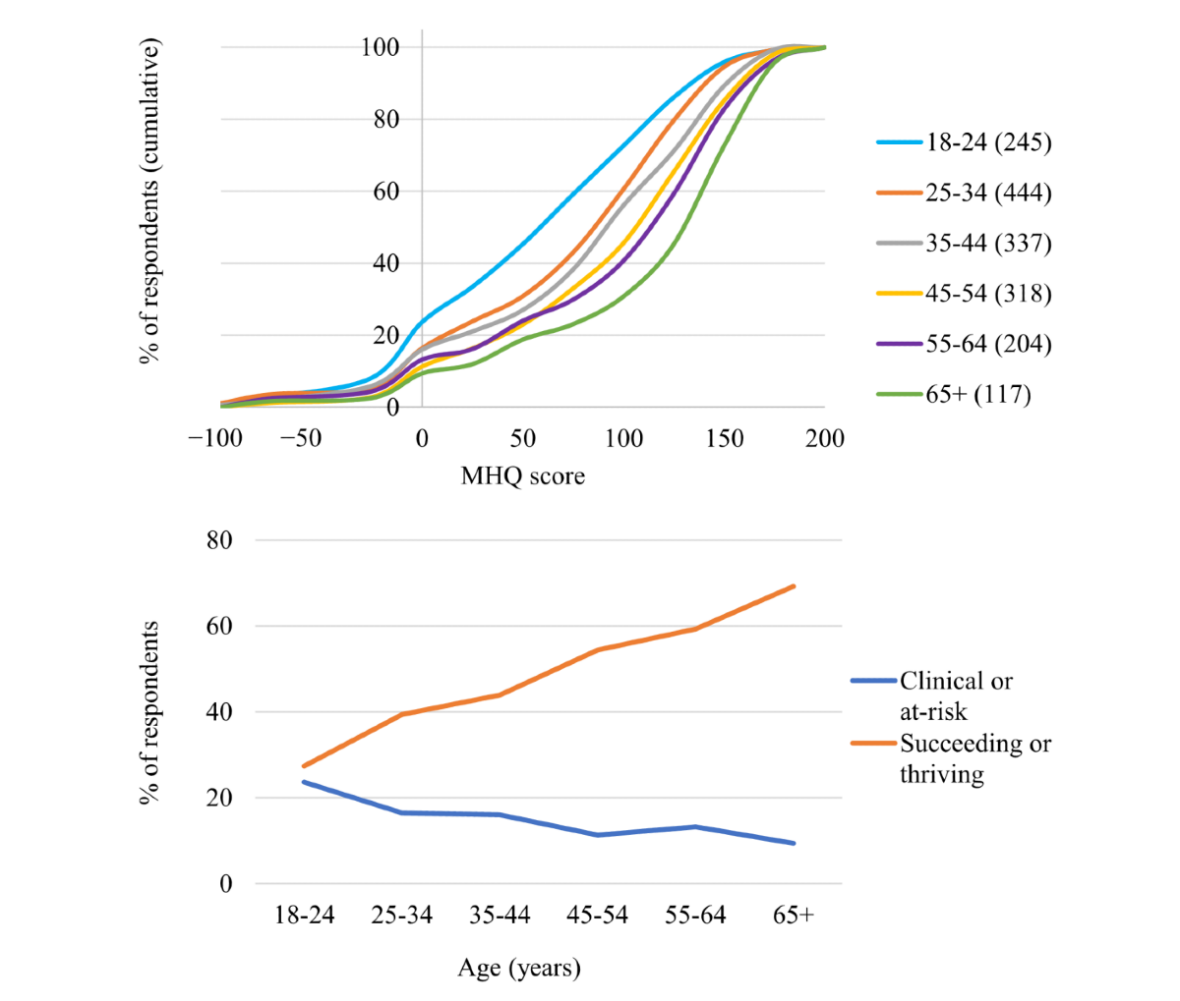
Distribution of Mental Health Quotient scores across ages. Shows both the cumulative percentage of respondents across the Mental Health Quotient score range for each age bracket (N values for each age bracket shown in the legend) and the linear increase in the proportion of succeeding or thriving (Mental Health Quotient scores above 100) and the decrease in the proportion of at-risk or clinical (Mental Health Quotient scores below 0) from younger to older age groups. MHQ: Mental Health Quotient.

Next, we show the initial results of overall MHQ scores by gender (Figure 6) and age (Figure 7). The distribution for males and females showed that a greater proportion of females reported negative MHQ scores compared with males (177/1018, 17.39% for females compared with 12.6% [77/609] for males; Figure 6), with the greatest difference being in the mood and outlook subcategory (204/1018, 20.04% of the female respondents were at-risk or clinical compared with 92/609, 15.1% of the male respondents), and mind-body (169/1018, 16.60% of the female respondents were at-risk or clinical compared with 44/609, 7.2% of the male respondents) subcategories. Both subcategories contain a large proportion of depressive symptoms; therefore, this finding is in line with the gender differences reported elsewhere [26-28]. In addition, MHQ scores differed substantially by age, with older age brackets having increasingly positive scores overall (Figure 7). MHQ scores of respondents in the 18 to 24 years age range were sharply lower, with 23.7% (58/245) in the negative at-risk or clinical range and only 27.3% (67/245) in the succeeding or thriving range. The proportion of respondents who were at-risk or clinical declined with age from 23.7% (58/245) to just 9.4% (11/117) in the 65+ years age group, and the proportion of those succeeding or thriving (ie, scores above 100) increased with age from 27.3% (67/245) to 69.2% (81/117). This pattern is in line with data from other sources [29]. This view by age and gender was not significantly different between respondents from the United States alone versus respondents from all other countries together. However, at this stage, because of the small representation from other countries (maximum of 149/1665, 8.95% for any individual country), a country-wise comparison was not possible.

#### MHQ Subcategory Scores

**Figure 8:**
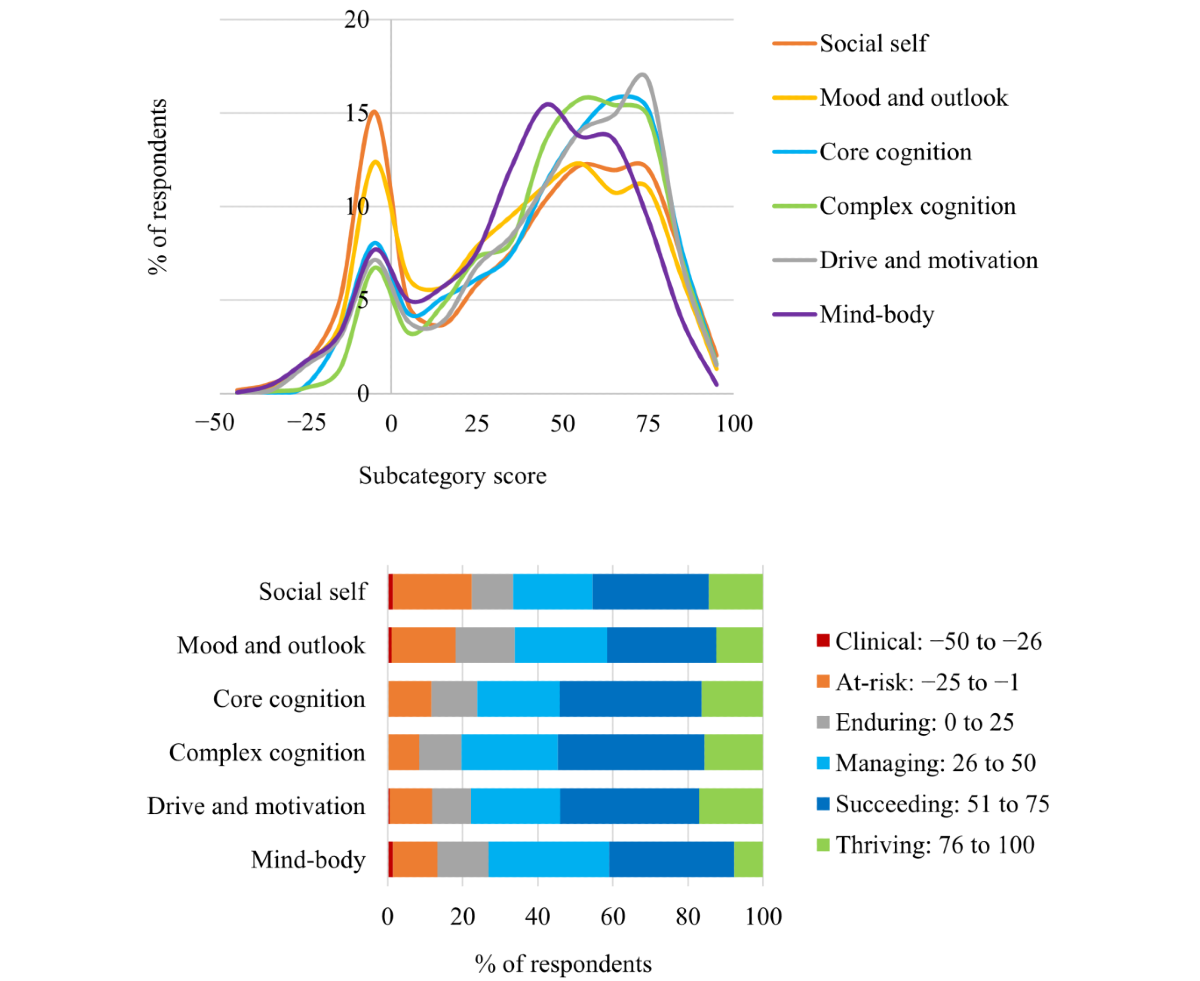
The distribution of Mental Health Quotient subscores for each of the 6 subcategories of mental health and the percentage of respondents for each of the 6 subcategories of mental health for each Mental Health Quotient score level. These levels are (from left to right) clinical, at-risk, enduring, managing, succeeding, and thriving. Numbers in the legend denote the Mental Health Quotient score range for each level.

Next, we show the distribution of MHQ subcategory scores across each of the 6 subcategories of mental health (Figure 8). The distribution structure is highly similar to the overall MHQ across all categories, with a normal distribution in the positive range and a skew in the negative range. The average values across the entire score range for each subcategory were as follows: core cognition 47 (median 54, mode 75); complex cognition 49 (median 53, mode 51); drive and motivation 47 (median 54, mode 74); mood and outlook 39 (median 43, mode −2); social self 39 (median 46, mode −1); and mind-body 40 (median 45, mode 65). Within the positive score range, the average, median, and modal values were as follows: core cognition 54 (median 57, mode 63); complex cognition 54 (median 56, mode 51); drive and motivation 55 (median 57, mode 74); mood and outlook 49 (median 51, mode 80); social self 53 (median 56, mode 75); and mind-body 48 (median 49, mode 65). A few key aspects warrant mention: the social self-subcategory, in particular, had a comparatively large proportion of people in the negative range (374/1665, 22.46% overall and 24/1665, 1.44% in the clinical range) followed by mood and outlook (302/1665, 18.14% overall, and 19/1665, 1.14% in the clinical range), indicating that challenges relating to these aspects of mental health were highly prevalent in the population of respondents (Figure 8). In contrast, the proportion of respondents facing serious challenges in their cognition (core and complex), drive and motivation, and mind-body were comparatively smaller.

## Discussion

### A New Tool for Assessing Individual and Population Mental Health and Well-Being

Assessment is the first step in identifying individuals and groups who are most at risk from mental health challenges as well as understanding the overall mental well-being of a population. However, existing mental health assessment tools exhibit several limitations [16] that hinder both effective transdisorder diagnosis and their application to the general population. Here, we present the MHQ, a uniquely designed web-based assessment tool that provides both an individual view of mental health and clinical risk and, when aggregated, a population view of overall mental well-being.

### MHQ as a Unique and Comprehensive View of Both Symptoms and Assets

The MHQ spans the breadth of mental health symptoms associated with major psychiatric disorders in a standardized and unbiased manner as well as assets and abilities important for overall mental well-being. The fact that 97.96% (1921/1961) of respondents found the MHQ easy to understand, and that it took, on average, only 14 min to complete, indicates that the tool is highly accessible to the general population.

The MHQ was uniquely developed based on an extensive review of symptoms from 126 assessment tools across 10 different mental health disorders as well as taking into account disorder agnostic approaches to mental health, such as RDoC [20-22]. In this regard, it represents the most comprehensive symptom profiling available, overcoming many limitations and biases of existing tools that include only partial lists of symptoms and are often skewed toward feelings or behaviors [16]. The MHQ also goes beyond a disorder-based approach (ie, a focus on negative symptoms alone) with the inclusion of spectrum items that consider a person’s mental abilities and assets. This aspect, rarely considered by existing mental health assessment tools, is critical to existing views of mental well-being [1] and addresses the growing realization that positive aspects of mental health are essential for an integrated view of health [2,30].

Together, this design approach allows respondents, on an individual level, to obtain a holistic picture of both concerns and abilities across their results profile, while at the population level, it ensures that insights are not based on an incomplete or biased picture of reported symptoms and functions.

### Insights Into Individual Mental Health

On the one hand, the MHQ can be used to provide personalized insight into an individual’s mental health in a manner that is disorder agnostic and avoids the ambiguity of disorder classification [18]. These insights are accompanied by feedback that is generated based on the individual scoring profile. This allows at-risk individuals to self-identify so that they can seek appropriate support before reaching clinical levels of distress or impairment. For example, in this preliminary data set, 13.09% (218/1665) of the respondents were identified as being at-risk, whereas 2.46% (41/1665) of the respondents likely required immediate clinical intervention, of which 95% (39/41) met the DSM-5 criteria for a mental health disorder. It also provides a mechanism for individuals within a normal healthy range to evaluate dimensions of their mental health and identify challenge areas so that they can take action (eg, make adjustments to their lifestyle) to strengthen and preserve their well-being even if they are not considered clinically at risk. Owing to its close equivalence to diagnostic outcomes based on DSM-5 criteria, the MHQ can also be used as a fast patient screen on admittance to a hospital clinic, where individual scores and mappings to DSM-5 disorder classifications, as shown in Table 1, can provide an initial impression of a patient’s symptoms and diagnosis to guide faster paths to treatment.

### Validation of the MHQ Against DSM-5 Diagnostic Criteria and Known Epidemiology

The preliminary data presented here from just 1665 adult respondents demonstrated that overall 15.56% (259/1665) of the respondents were identified as being at-risk (218/1665, 13.09%) or requiring immediate clinical intervention (41/1665, 2.46%). Comparisons of MHQ scores against DSM-5 criteria also revealed a low false-positive rate (2/41, 5%) within the clinical score range, where 95% (39/41) of the respondents met the criteria for a diagnosis of at least one mental health disorder. There was also a low false-negative rate (16/1406, 1.14%) within the positive score range (from enduring to thriving), indicating that 98.86% (1390/1406) of respondents with a positive MHQ score did not meet the criteria for a mental health disorder diagnosis. The close alignment between MHQ scores and the degree to which people meet DSM-5 diagnostic criteria demonstrates its validity as a mental health assessment tool capable of identifying at-risk individuals within a population as well as providing a comprehensive cross-disorder clinical view.

One limitation was that the MHQ mapping to the DSM-5, and the subsequent diagnostic indication, only took into account the severity of symptoms and not the duration or frequency of symptoms required for some disorders, as these aspects do not form part of the MHQ. However, the MHQ is also able to identify those people with a large number of severe clinical symptoms in need of help, whose symptoms do not fall specifically into any particular disorder classification.

At the population level, the proportion of respondents reporting negative scores is in line with annual prevalence rates of mental health disorders reported from other sources [23-25]. In addition, female respondents scored slightly more poorly, especially in the mood and outlook and mind-body subcategories, both subcategories with a large proportion of depressive symptoms, in line with gender differences reported elsewhere [26-28]. Finally, the data showed that individuals within the youngest age bracket (18-24 years) were most at risk of experiencing mental health challenges, which is also in line with data from other sources [29]. Thus, the overall results of the MHQ are in line with other epidemiological estimates along various dimensions, demonstrating its validity as an epidemiological mental health assessment tool.

### Potential Applications of the MHQ

The MHQ was designed to be easy to implement in research initiatives using large populations of individuals to obtain insights into the profile of mental health challenges and positive well-being. When used in a large-scale epidemiological context, relating MHQ scores to a range of demographic, experiential, and situational variables can support the development of relevant interventions or policies that could induce larger-scale shifts in population well-being. Furthermore, the MHQ can be used within specific organizations, such as companies or universities, to measure and track the overall mental health and well-being of their workforce or student body, respectively; to support the design of tailored interventions suited to that specific group; to identify at-risk individuals or subgroups; and to assess the impact of any support programs. The MHQ can also be used in a clinical context as a first-line screening tool within both primary care and psychiatric clinics. From a research perspective, the results obtained from the MHQ can also enable a better understanding of the relationship between individual symptoms and symptom profiles and underlying biomarkers and be used to examine the efficacy of new treatment regimes.

### Identifying the Borders Between Abnormal and Normal Mental Health

The development of an assessment tool that covers the breadth of mental ill health through to positive functioning, and one that is accessible to the general population, is also relevant for one of the major discussion points pertaining to the diagnosis and classification of mental disorders, namely, the distinction between normal and abnormal mental health [12,14,15]. As most negative mental states, such as sadness, despair, anxiety, fear, agitation, and anger, are not abnormalities per se but normal responses to life’s ups and downs, being able to decipher whether a person is responding normally to difficult circumstances, or experiencing pathological levels of distress or impairment, is not straightforward [13]. One challenge underpinning this debate relates to the fact that, currently, there is a poor understanding of the state and diversity of mental health across a normal population. Thus, if there is a poor understanding of what the continuum of normal mental health looks like, how can we understand when it is starting to slide into abnormal. Such a distinction is necessary not only to prevent false positives in diagnosis, a label that can be unduly associated with stigma but also to ensure that people receive appropriate treatment and that clinical research studies investigating underlying etiologies select from appropriate sample pools. The MHQ assessment tool has been constructed to capture this breadth of function from positive assets to extreme distress to establish these distinctions.

Psychiatric disorders are among the most disabling health conditions worldwide, creating a significant burden on individuals and societies [31]. Assessments of mental health that are accessible to the general population support the early identification of at-risk individuals or subgroups and reveal relevant risk factors. This, in turn, can help reduce the burden by facilitating the development of relevant and effective interventions and policies before symptoms escalate to clinical levels. The importance of population accessible tools is further emphasized by the reported gap between those with severe distress and impairment and those receiving the help and support they need [32]. The MHQ aims to help realize the vital goals of mental health prevention and support by providing a means to measure and track population mental health. Going beyond this, the MHQ ultimately seeks to enable a paradigm that can manage and improve the lives and well-being of all people, and not just those with a clinical disorder.

## Abbreviations

ADHD: attention-deficit/hyperactivity disorder
ASD: autism spectrum disorder
DSM: Diagnostic and Statistical Manual of Mental Disorders
ICD: International Classification of Diseases
MHQ: Mental Health Quotient
OCD: obsessive-compulsive disorder
PTSD: post-traumatic stress disorder
RDoC: Research Domain Criteria
